# Keep the Mouth Closed

**Published:** 1890-12

**Authors:** 


### KEEP THE MOUTH CLOSED.

The following is suggested to snorers : There are two channels in which the air travels in going to the lungs, namely, the nose and mouth. These two passages unite in a common cavity, and from that point there is but one tube leading to the lungs. There is a bone called the hard palate, which forms the roof of the mouth and the floor of the nose, separating these two air channels from each other. At the inner or posterior end of the bone is a little body called the soft palate, made of muscle and covered with a delicate skin. This soft palate is attached at one end to the hard palate ; the other end hangs loose and moves or laps in the act of breathing, something like a window curtain when acted upon by a current of air. This is its condition while we are asleep or awake, though during sleep it lacks in tonicity, being much more relaxed or flabby than when we are awake. Now, in order to snore, one must keep the mouth open as well as the nose, and in this condition the two currents of air passing in and out together during the act of breathing catch this little curtain between them and throw it into rapid vibration. This vibration, more or less intense and sonorous, is what we call snoring.

It is only with the mouth open that snoring can be accomplished during sleep. Awake if the nose is closed by the thumb and finger, by taking a forcible breath, it is possible to snore, and the same result may be accomplished with the mouth shut and the nose open ; but the muscular effort necessary to its accomplishment is more than we can command during sleep, and would wake up the person who might unconsciously make the effort.

